# Physicochemical differences between camelid single-domain antibodies and mammalian antibodies

**DOI:** 10.55730/1300-0152.2676

**Published:** 2023-12-07

**Authors:** Nazlı Eda ESKİER, Doğa ESKİER, Esin FİRUZAN, Sibel KALYONCU UZUNLAR

**Affiliations:** 1 İzmir International Biomedicine and Genome Institute, Dokuz Eylül University, İzmir, Turkiye; 2 İzmir Biomedicine and Genome Center, İzmir, Turkiye; 3 Department of Statistics, Faculty of Sciences, Dokuz Eylül University, İzmir, Turkiye

**Keywords:** Antibody, nanobody, camelid, physicochemical properties, logistic regression, machine learning

## Abstract

**Background/aim:**

In recent years, single-domain antibodies, also known as nanobodies, have emerged as an alternative to full immunoglobulin Gs (IgGs), due to their various advantages, including increased solubility, faster clearance, and cheaper production. Nanobodies are generally derived from the variable domain of the camelid heavy-chain-only immunoglobulin Gs (hcIgGs). Due to the high sequence homology between variable heavy chains of camelids (V_H_Hs) and humans (V_H_s), hcIgGs are ideal candidates for nanobody development. However, further examination is needed to understand the structural differences between V_H_s and V_H_Hs. This analysis is essential for nanobody engineering to mitigate potential immunogenicity, while preserving stability, functionality, and antigen specificity.

**Materials and methods:**

We obtained the V_H_ and V_H_H sequences of various camelid and non-camelid mammalian antibodies from public databases and used multiple sequence alignment based on the Chothia numbering scheme. Aligned sequences were subjected to diverse analyses encompassing paratope length, binding prediction, motif, disulfide bridge, salt bridge profiling, and physicochemical characteristic distribution. Logistic Regression coupled with the Boruta - Random Forest algorithm facilitated the comprehensive examination of physicochemical properties.

**Results:**

Our findings revealed longer, less variable paratope sequences in V_H_Hs, along with specific antigen binding residues with increased binding potential compared to V_H_s. Although the V_H_s showed more heterogeneous noncanonical disulfide bond patterns, the V_H_Hs had a higher number of noncanonical disulfide bridges. Intriguingly, a typical salt bridge between the 94th and 101st positions in the V_H_s had a very low encounter rate in the V_H_Hs. Surprisingly, we also identified notable differences in the physicochemical patterns of mostly conserved frameworks (FWs), especially the FW2 and FW3 regions, between V_H_s and V_H_Hs.

**Conclusion:**

Our findings point to possible key sites in V_H_Hs as candidate residues for nanobody engineering efforts.

## 1. Introduction

Antibodies play a vital role in safeguarding our immune system. Among these immune defenders, immunoglobulin Gs (IgGs) are the most commonly found ones in humans and mammalians (Vidarsson et al., 2014). However, different species possess different antibody formats with novel benefits that contribute to an increased survival rate. Camelids have evolved a solution to cope with extreme environments through adaptive changes (Flajnik et al., 2011), and such environmental circumstances eventually paved the way for fully functional alternative antibodies: heavy-chain-only IgGs (hcIgGs) (Hamers Casterman et al., 1993).

**Figure 1:**
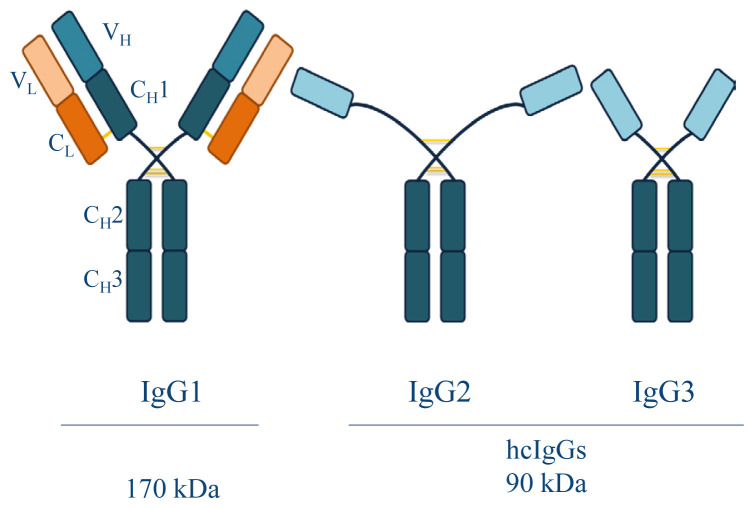
Different antibody types found in camelids. Light orange sections show the variable light chain (V_L_) domain, while the light blue sections represent the variable heavy chain (V_H_). Dark orange sections and dark blue regions indicate the constant region of the light chain (C_L_) and the constant regions of the heavy chain (C_H_1–3), respectively. Yellow lines indicate intradomain disulfide bridges.

To generate diversity in variable domains, members of the Camelidae (camels, llamas, alpacas, and vicunas) can encode both conventional IgGs and hcIgGs (Figure 1) (Muyldermans et al., 1994). Approximately the same amount of heterodimeric canonical IgGs (IgG1) and homodimeric hcIgGs (IgG2 and IgG3) coexist in the camelid repertoire, even sharing the same germline (D) J-genes during B-cell maturation (De Genst et al., 2006). hcIgG molecules lack the light chain (L) and the first constant region of the heavy chain (H). To compensate, hcIgGs developed unique adaptations, such as a longer hinge region, which confers physical flexibility upon antigen binding (Muyldermans, 2013). There are unconventionally charged and polar residues at the former variable H–variable L (V_H_–V_L_) interface, which correspond to the framework (FW)-2 region in hcIgGs. These preclude association with other hcIgG molecules and increase solubility and stability (Goldman et al., 2017). These FW2 residues are extremely conserved in both IgGs and hcIgGs (Mitchell and Colwell, 2018). Although there are six different complementarity-determining regions (CDRs) (HCDR1–3 and LCDR1–3) responsible for antigen binding in the IgGs, there are only HCDR1–3 in hcIgGs (Vu et al., 1997). Instead of relying on the LCDRs for epitope recognition, hcIgGs have circumvented their absence by recruiting more residues into HCDR1 and HCDR3 (Muyldermans et al., 1994). The HCDR3 loop in the variable heavy chains of camelids (V_H_Hs) shields the FW2 residues to preclude the interaction of hydrophilic residues found at the former V_H_-V_L_ association site (Bond et al., 2003). To maintain the stability of such loops, the introduction of internal disulfide bonds between HCDRs is another adaptation in the format of hcIgGs. The presence of noncanonical disulfide bonds further improves the stability by allowing reversible refolding of the structure as well as reducing the entropic folding penalty, while providing protection against proteases under physiological conditions (Mendoza et al., 2020). Intriguingly, through all these adaptations, hcIgGs retain the same level of antigen binding capability as IgGs and might surpass conventional IgGs with superior affinity and avidity (Nguyen et al., 2002).

The field of antibody research has witnessed a paradigm shift with the emergence of stand-alone V_H_Hs of hcIgGs, also referred to as nanobodies or single-domain antibodies. The term “nanobody” was first coined because they are stable and soluble binders, retaining their nanomolar affinity to their target even at the smallest possible size. Nanobodies possess several advantages, such as rapid clearance, robustness, cheaper and faster production, resistance to aggregation, improved stability and solubility, recognition of novel/hidden epitopes, faster extravasation, and better tumor/tissue penetration compared to IgGs (Pillay and Muyldermans, 2021). Due to these benefits, potential applications of nanobodies have spread across many different fields, such as therapeutics, diagnostics, in vivo imaging, and even agriculture (Muyldermans, 2021).

While monoclonal antibodies have been the primary focus of optimization efforts, there is growing interest in optimizing other antibody formats. There are engineering approaches to convert traditional IgGs into nanobody formats. Camelization is a process where the monomeric human variable heavy chain (V_H_) is used as a scaffold and key residues of V_H_Hs are introduced into this moiety (Riechmann and Muyldermans, 1999). The V_H_Hs of hcIgGs display 80% sequence homology and structural similarity to human V_H_s (Conrath et al., 2001). Due to this homology between camelids and humans, V_H_s are ideal candidates for nanobody development (Bélanger et al., 2019).

Although prior studies have acknowledged differences between V_H_s and V_H_Hs, the precise and comprehensive structural and physicochemical characterization of these variances remains insufficiently explored. While it is widely recognized that V_H_Hs possess longer CDR3 regions, the exact lengths and their functional implications have yet to be precisely determined. Additionally, the physicochemical nature of conserved FW regions and the prevalence and precise locations of noncanonical cysteine bonds in V_H_Hs remain elusive.

## 2. Materials and methods

### 2.1. Datasets

**Figure 2:**
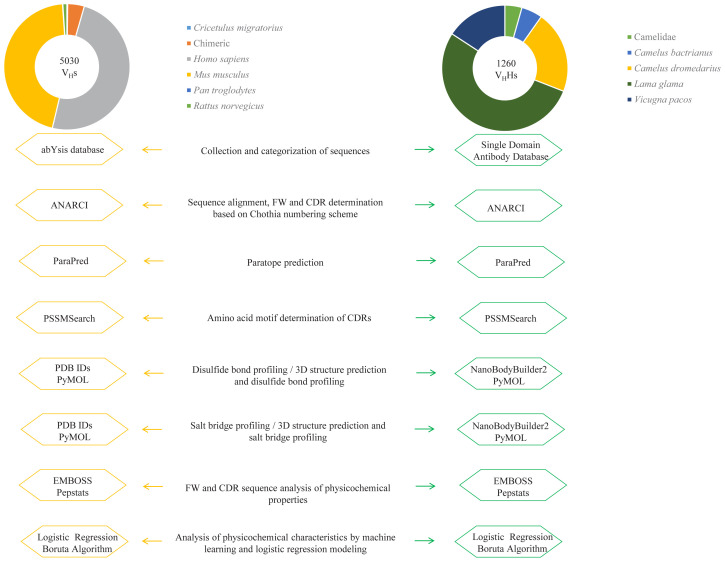
Flowchart showing the analyses conducted in this study and the tools used for each analysis. The pie charts indicate the fraction of each species within the obtained sequences, with the total number of sequences shown inside the circle.

In this study, we analyzed the V_H_ sequences of 5030 mammalian IgGs (*Cricetulus migratorius*; chimeric *Homo sapiens*/*Mus musculus*; chimeric *Mus musculus*/*Homo sapiens*; *Homo sapiens*; *Mus musculus*; *Pan troglodytes*; *Rattus norvegicus*) acquired from the abYsis database (Swindells et al., 2017) and the V_H_H sequences of 1260 nanobodies (camelid otherwise unspecified; *Camelus bactrianus*; *Camelus dromedarius*; *Lama glama*; *Vicugna pacos*) publicly available in the Single Domain Antibody Database (Wilton et al., 2018). We obtained the V_H_ sequences by trimming non-V_H_ domains from the given sequences. The list of V_H_s and V_H_H IDs is available in Supplementary File 1. After obtaining the sequences, we conducted numerous in-depth analyses (Figure 2).

### 2.2. Multiple sequence alignment

To ensure a precise and standardized annotation of HCDRs and FW regions, and to facilitate a comprehensive and uniform analysis of the aligned sequences, we aligned multiple FASTA sequences according to the Chothia numbering scheme using a locally installed version of the ANARCI Antigen Receptor Numbering and Receptor Classification tool (Dunbar et al., 2016) to define HCDRs and FW regions (Table S1).

### 2.3. Paratope binding prediction

After the determination of HCDRs, to categorize the binding potential differences between the CDRs of V_H_s and V_H_Hs, we predicted the paratope binding regions and binding scores including those for the two flanking residues of HCDRs using “Parapred: Antibody Paratope Prediction Using Convolutional and Recurrent Neural Networks” (Liberis et al., 2018), which is compatible with the output of ANARCI.

### 2.4. Sequence conservation motif analysis

To elucidate conserved amino acids at specific positions within the sequences in a comprehensive and visually intuitive manner, we determined the amino acid motifs of V_H_ and V_H_H sequences using WebLogo (version 2.8.2) (Crooks et al., 2004), an online application for visualization of protein motifs, to show which amino acids are conserved at certain positions. FW and HCDR motif visualization is based on an amino acid frequency scoring method.

### 2.5. Disulfide bond analysis

We aligned all V_H_ and V_H_H sequences that harbored double or multiples of two cysteine residues according to the Chothia numbering system. We highlighted canonical/noncanonical disulfide bridges and confirmed them both sequentially and structurally using PyMOL software (version 2.5.5) (Deac et al., 2019). Many V_H_Hs lack PDB IDs, as most of the sequence information was obtained from publications. To allow for the structural modeling of V_H_Hs even in instances where PDB IDs were unavailable, we homology-modeled the 3D structures of the V_H_Hs using NanoBodyBuilder2 (Abanades et al., 2023) to produce the structural modeling of the V_H_Hs with known sequence information.

### 2.6. Salt bridge analysis

After the previous analyses, to better understand the electrostatic interactions that contribute to the structural stability and functionality of the aligned V_H_ and V_H_H sequences, we detected salt bridges in sequences aligned according to Chothia numbering. Specifically, we filtered the V_H_ and V_H_H sequences to identify the presence of R, K, and H residues at the 94th position, as well as the D and E amino acids at the 101st position.

### 2.7. Physicochemical properties determination

We used the EMBOSS Pepstats tool (Madeira et al., 2022) to facilitate the calculation of physicochemical contents for both HCDRs and FW regions and to obtain information regarding the amino acid composition, hydrophobicity, charge, and other physicochemical properties as listed in Tables S2 and S3.

### 2.8. Statistical analysis

To better determine the power of the physicochemical variables to discern between V_H_s and V_H_Hs, we applied a logistic regression modeling approach and the Boruta algorithm based on supervised machine learning to data produced by EMBOSS Pepstats (Supplementary File 2).

### 2.9. Tools and plots

We ran all Unix-compatible tools on the Windows operating system and accessed them through a Docker Container. We generated length variation bar plots and antigen binding prediction score heatmaps using the R statistical computing environment (version 4.0.0), using the *ggplot2* graphics package (version 3.4.2) and the *pheatmap* (version 1.10.12) heatmap generation package, respectively. We conducted binary logistic regression using the *pscl*, *caret*, and *car* R packages to measure the statistical significance of physicochemical variables (predictors) in the classification of V_H_s (0) and V_H_Hs (1). We visualized the Boruta algorithm via the Boruta R package results using the *ROCR* R package. Further methods can be found in the Supplementary Files.

## 3. Results and discussion

### 3.1. Length variation in HCDRs

**Figure 3:**
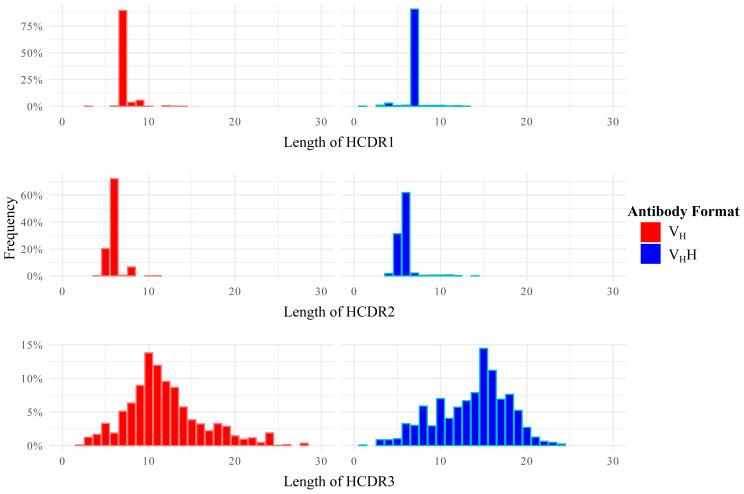
Bar plot displaying the HCDR length variation of VHs and V_H_Hs for HCDR1–3 and their encounter frequencies in each dataset of VHs and V_H_Hs. VHs are shown in red, while V_H_Hs are shown in blue. The y-axis represents the frequency of HCDR length based on their encounter rates. The HCDR windows were defined according to the Chothia numbering scheme.

Initially, we performed a comparative analysis of the differences in the lengths of HCDRs (Figure 3). The distribution of HCDR1 and HCDR2 length profiles were highly similar between V_H_s and V_H_Hs. As anticipated, the most significant variation in length was encountered in HCDR3, which is the region primarily responsible for target binding. The length distribution of V_H_s was skewed to the left, with a mode length of 10 residues. Conversely, the V_H_Hs exhibited a length distribution that was skewed to the right, with a mode of 15 residues, consistent with other studies that highlighted the prevalence of longer HCDR3 loops in V_H_Hs. Interestingly, while V_H_s had a low number of outlier sequences with longer HCDR3s, the overall count of HCDR3s longer than 15 was higher in V_H_Hs than in V_H_s. There are two main functions of typically longer HCDR3s in V_H_Hs: (i) to fold over the hallmark residues residing at FW2, shielding those hydrophilic residues to preclude them from any other interactions, and (ii) to increase the antigen specificity/affinity by enlarging the surface to compensate for the absence of LCDRs (Conrath et al., 2005). The presence of longer HCDR3s became apparent when calculating average HCDR lengths based on species (Table S4 and Table S5). When examining the species-specific HCDR lengths, we found that *Vicugna pacos* exhibited the shortest average V_H_H HCDR3 length, followed by *Camelus dromedarius* (Table S5). These species-specific variations in the antibody repertoire can stem from the specific immune responses they generate. These differences can arise from evolutionary adaptations, such as variations in germline gene usage, or selection pressures acting on the antibody repertoire.

### 3.2. Antigen binding scores

**Figure 4:**
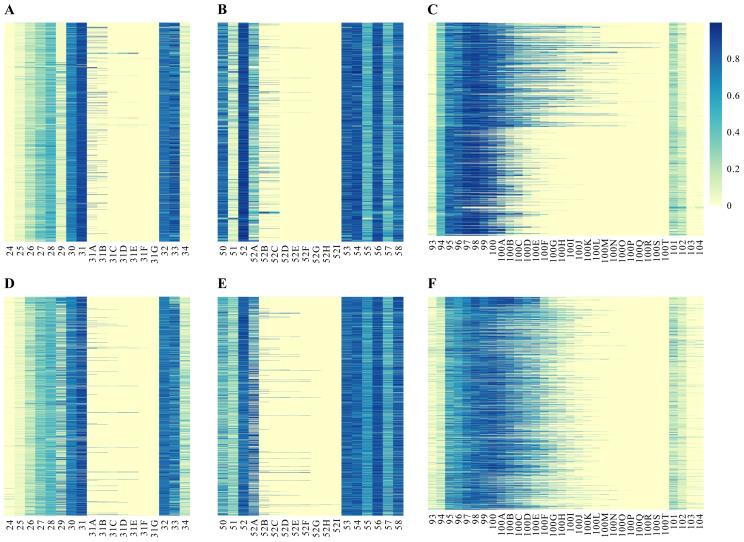
Heatmaps displaying the antigen binding prediction scores of HCDR1–3 regions of VHs (A–C) and V_H_Hs (D–F). Multiple sequences were aligned based on the Chothia numbering scheme. High binding scores are denoted by blue cells, whereas low binding scores are represented by yellow cells. The flanking framework residues on the two leftmost and rightmost columns serve as predictors for the binding score generated by the software. To facilitate proper alignment between VHs and V_H_Hs, empty columns were intentionally inserted for the alignment of shorter loops, all of which are indicated by yellow cells at alternating positions.

CDRs are the most important parts in the antibody structure, as they mostly define the specificity/affinity to the target antigen. To identify which HCDR residues are the most important in target recognition, we proceeded to analyze the paratope scores indicating antigen binding within the HCDRs and the flanking FW regions (two residues from each side of the HCDR loops). According to the heatmaps, the V_H_s and V_H_Hs exhibited similar antigen binding profiles (Figure 4). Notably, HCDR1 exhibited the lowest contribution to antigen binding ability (Figures 4A and 4D). In each case, the 31st position consistently yielded the highest sum of scores, underscoring its pivotal role as the most important residue within HCDR1. The extra residues featured at the 31st A–G positions consistently exhibited high binding scores, suggesting their significant role in antigen binding whenever they were incorporated into the HCDR1 sequence. The 32nd position, as the last residue of HCDR1, and the 33rd position, as the first residue of FW2, also showed very high binding scores.

In HCDR2, the 52nd position had the highest binding score, closely followed by the 54th and 56th residues. Notably, the 52nd A residues were prevalent in numerous sequences within both datasets. In V_H_s, the presence of extra residues at 52A–F was associated with moderately to highly elevated binding scores (Figure 4B). For V_H_Hs, HCDR2 sequences incorporating a residue between the 52A–I positions consistently had very high binding scores (Figure 4E). These observations suggest that the inclusion of an additional residue within the frame of the 52nd position plays a significant role in antigen binding.

As anticipated, HCDR3 exhibited the greatest variability in paratope residues. For V_H_s, the 94th position made a considerable contribution (Figure 4C). However, in V_H_Hs, the functionality of the 94th residue was less pronounced (Figure 4F). Notably, in V_H_s, there was a high-intensity concentration of binding scores within the range of 95–100A. Conversely, when examining the V_H_Hs, this intensity concentration shifted towards the C-terminus, with the highest-scoring residues clustered between the 97 and 100B positions.

### 3.3. Differences between conserved motifs

**Figure 5:**
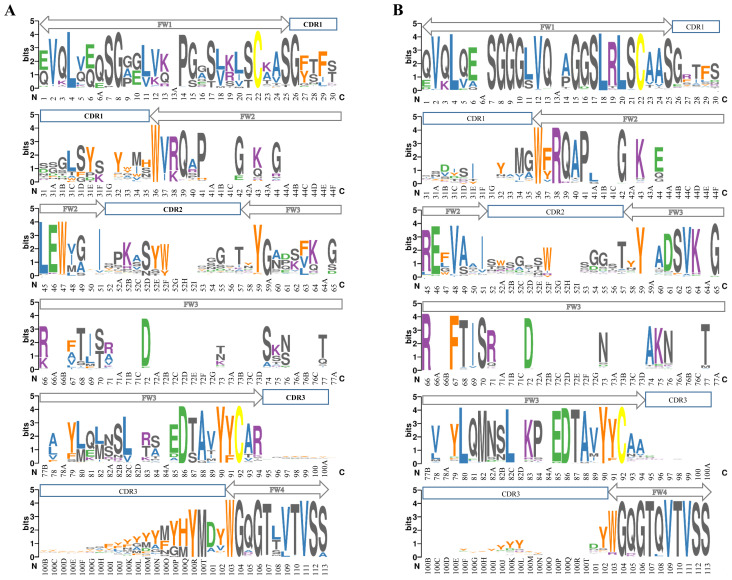
Graphical logo representations of multiple sequence alignment of the complete sequence datasets of (A) VHs and (B) V_H_Hs. The multiple sequences were aligned based on the Chothia numbering scheme. The size of each logo reflects the relative frequency of amino acids at each position, measured in bits. Color codes were applied based on single-letter-code amino acid biochemical properties; basic: K, R (purple); acidic: D, E (green); aliphatic: A, I, L, V (blue); aromatic: F, H, W, Y (orange); cysteine: C (yellow); mostly small: G, M, N, P, Q, S, T (dark gray). Certain amino acids were categorized in multiple subclasses. The grey and dark blue boxes indicate the borders of the FW and HCDR regions, respectively.

Next, we extended our investigation to conservation motif analysis, which involves systematic examination of FWs and HCDRs, providing a holistic view of the sequence conservation landscape for both V_H_ and V_H_H sequences (Figure 5). As CDRs exhibit a high degree of variability depending on their target specificity, direct comparisons between them were often not meaningful. While interpreting the results of this analysis, we concentrated on the comparatively more conserved FW regions rather than the hypervariable HCDRs.

In FW1, the remarkable distinction between V_H_s and V_H_Hs lay primarily in their initial amino acid residues. Most V_H_Hs started with ‘Q’ instead of ‘E’ compared to their V_H_ counterparts. In FW1, V_H_s exhibited standout basic residues at the 9th, 10th, 12th, and 13th positions, resulting in a positive charge. Specifically, the 23rd position in the V_H_s presented a positively charged ‘K,’ while V_H_Hs displayed more conserved ‘A’ in addition to ‘K.’

A prominent divergence occurred in FW2 at the 37th, 44th, 45th, and 47th positions, and these transitions are well-known hallmark residues in camelids. The ‘VGLW’ motif at these hallmark residue positions in V_H_s underwent a transformation into ‘FERF/YERF’ in V_H_Hs. There were other minor differences at the 48th and 49th positions, although not as obvious as the hallmark residues.

The number of positions with minor differences between V_H_s and V_H_Hs notably increased in FW3, namely at the 63rd, 71st, 73rd, 74th, 78th, 81st, 82nd, 83rd, 84th, and 94th positions. The 94th position majorly differentiated between V_H_s and V_H_Hs, with V_H_s featuring ‘R,’ a positively charged amino acid, in contrast to ‘A’ in V_H_Hs.

Hydrophobic residues have a natural tendency to avoid contact with solvents and tend to fold inward, contributing to the protein’s structural stability. By minimizing the hydrophobic content in the constant regions, V_H_Hs promote proper folding and overall stability of the antibody structure. Considering the results of the physicochemical properties of FW2 and FW3, the prevalence of hydrophilic residues in V_H_Hs compared to V_H_s suggests a potential indicator for the higher stability and solubility of V_H_Hs, even in the absence of a V_L_ (Mitchell et al., 2018). In addition to FW2, our findings highlight the critical role of these FW3 residues, underscoring their potential impact on the overall structure and function of the antibody. As a prospective avenue, researchers can explore the differences identified in this study, especially in these regions, to guide future investigations.

FW4 represents the relatively shorter region, and it is the most conserved segment within the entire variable domain in terms of sequence. However, there were notable differences in our dataset. V_H_s predominantly recruited aromatic ‘Y’ or ‘W’ residues around the 100th position, highlighting a consistent feature in their HCDR3 region. This preference for aromatic residues in V_H_s may play a crucial role in their antigen-binding specificity and affinity. Another intriguing observation within our dataset was the substitution of the ‘W’ at the 103rd position in the FW4 of V_H_Hs. At the 108th position, the ‘Q’ in V_H_Hs transitioned into ‘L/T/S’ in V_H_s.

Moreover, similarly consistent patterns emerged in both the V_H_ and V_H_H datasets once we clustered them into species-specific subsets (Figure S1 and S2). However, we observed conserved residues at the subset levels, which varied marginally from the overall consensus, since some species datasets contained relatively smaller numbers of sequences.

Our observations on the prevalence of differences among all FWs stand out, particularly given the emphasis in previous studies on the FW2 region as the primary locus for engineering the V_H_–V_L_ interface of antibodies to convert them into V_H_H format.

### 3.4. Encounter rate of noncanonical cysteine bonds

The introduction of a noncanonical disulfide bridge in addition to the canonical 22_nd_C–92_nd_C bond can further enhance the thermal stability of V_H_Hs. This additional disulfide bond can increase the thermal denaturation temperature, making the antibody more resistant to unfolding and improving its overall stability while helping to reduce the entropic folding penalty (D’Huyvetter et al., 2017). The presence of such extra bonds remedies the destabilizing effect caused by hallmark residues at FW2 and rigidifies the inherently longer HCDR3 loop by rendering a stronger, more specific interaction with their target (Govaert et al., 2012). These extra bonds could serve as structural reinforcements that contribute to the maintenance of the specific conformation required for effective antigen recognition and binding, particularly in antibodies with extended HCDR3 regions.

To analyze their contributions to antibody stability in different formats, we conducted a comprehensive exploration of the noncanonical disulfide bridges within antibody structures. We performed a dual analysis, encompassing both the examination of amino acid sequences and the inspection of 3D structural conformations. This combined approach allowed us to pinpoint the specific cysteine residues involved in the formation of noncanonical disulfide bridges in V_H_s. By combining the insights gained from sequence and structural analyses, we attempted to unravel the presence and significance of noncanonical disulfide bridges in both V_H_s and V_H_Hs.

**Figure 6:**
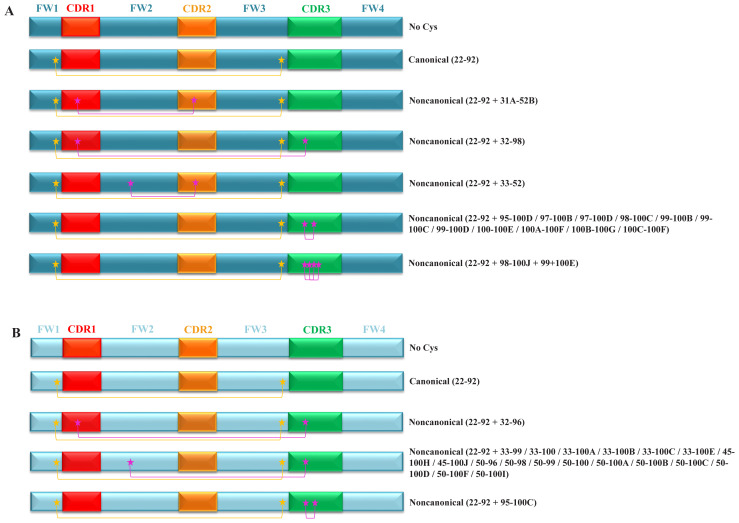
Canonical and noncanonical disulfide bond profiles of (A) VHs and (B) V_H_Hs. The FW regions (dark blue: VHs, light blue: V_H_Hs) and HCDRs (HCDR1: red, HCDR2: orange, HCDR3: green) are represented by boxes. Canonical disulfide bonds formed between 22ndC and 92ndC are denoted by yellow stars connected by a yellow line. Noncanonical cysteine bonds are represented with magenta stars joined by a magenta line. The shapes of FWs and HCDRs are not to scale. The positioning of the stars representing canonical and noncanonical cysteine residues serves as a putative visual representation and does not reflect their precise locations within the marked regions.

Our results showed that while V_H_s may have a variety of noncanonical cysteine types, V_H_Hs tended to have a higher frequency of these additional bonds, highlighting a distinctive structural characteristic in V_H_Hs (Figure 6). The encounter frequency of noncanonical bonds of V_H_s (2.6%) (Table S6) was significantly lower than that of V_H_Hs (5.5%) (Table S7). Among the observed noncanonical bonds in the V_H_s, four distinct types were identified, connecting various regions. These included bonds between HCDR1 and HCDR2, HCDR1 and HCDR3, FW2 and HCDR2, and HCDR3 and HCDR3 (Figure 6A). Our analysis of the V_H_Hs unveiled three distinct categories of noncanonical cysteine pairs coexisting alongside the canonical bond. These were formed between HCDR1 and HCDR3, FW2 and HCDR3, and HCDR3 and HCDR3 (Figure 6B). Although the frequency of V_H_ noncanonical bonds was lower than that of the V_H_Hs, the variety in their positions was higher than that of the V_H_Hs. For both formats, HCDR1–HCDR3 and HCDR3–HCDR3 noncanonical disulfide bonds were common. Bonds involving HCDR3s likely enhance the stabilization of HCDR3 loops, thereby contributing significantly to the structural integrity of the antibody’s antigen-binding site.

Notably, species-specific extra bonds were observed in both datasets (Tables S6 and S7). *Camelus dromedarius* stood out by demonstrating the highest diversity in terms of extra bonds occurring at various locations compared to other species in the dataset. In this study, 2.4% of *Lama glama* V_H_Hs were found to have noncanonical extra cysteine bonds (Table S7). *Homo sapiens* exhibited a relatively high frequency of noncanonical bonds, albeit at a rate of 5.3% (Table S6). *Vicugna pacos* and *Camelus dromedarius* exhibited the shortest average V_H_H HCDR3 length (Table S4), and these two species also had the highest occurrence of extra cysteine bonds (~10%) in their V_H_Hs (Table S7).

While some antibodies possessed remarkably long HCDR3 regions, extending up to 20 amino acids, it is essential to emphasize that length alone did not guarantee the presence of additional stabilizing bonds. The formation of disulfide bridges relies on the correct pairing of cysteine residues and their proper alignment in the protein’s three-dimensional structure. Failure to form disulfide bonds at these specific positions could be due to structural constraints, steric hindrance, or the absence of suitable cysteine pairing partners. For future prospective studies, researchers should delve deeper into the structural determinants influencing disulfide bridge formation, offering avenues to enhance antibody stability and functionality.

### 3.5. Stability-enhancing salt bridge profiles of V_H_s and V_H_Hs around HCDR3

Salt bridges are another mechanism used to regulate the folding and stability of antibodies. The 94th position had a high number of conserved ‘R’ residues and a positively charged amino acid in V_H_s, compared to the large number of ‘A’ residues in V_H_Hs as seen from motif conservation analysis (Figure 5). The presence of a positively charged residue (‘R’ or ‘K’) at this position, which serves as an N-flanking residue for HCDR3, enables the antibody to engage in favorable electrostatic interactions with negatively charged regions on the antigen, enhancing binding specificity. This formation not only influences the overall structure but also plays a pivotal role in mediating the interaction between antibodies and antigens (Tsumoto et al., 1996). As such, we performed a comprehensive comparison of the salt bridges in V_H_s and V_H_Hs. The presence of a salt bridge was a prevalent characteristic in V_H_s, typically observed with an ‘R’ residue at the 94th position and a ‘D’ residue at the 101st position (hereafter referred to as the canonical salt bridge, while other combinations of ‘R/H/K’ at the 94th position with ‘D/E’ at the 101st position, excluding the canonical pair, are considered noncanonical salt bridges).

There are several rule-determination studies suggesting the existence of a salt bridge between the 94th R and 101st D, yet they all used a narrow number of samples in their datasets, which is insufficient to claim that these particular residues are conserved at those positions. They specifically reported that the presence of a negatively charged 101st D residue consistently forms a salt bridge with the basic 94th R residue (Shirai et al., 1999; Weitzner et al., 2015). So far, these published studies have strictly focused on the 94th R–101st D interaction; however, a salt bridge in proteins is formed when two residues with opposite charges come together. In this context, the presence of a salt bridge is determined by the proximity of certain atoms. If the carboxylate of the ‘D/E’ side-chain is within 4.0 Å of an ammonium of an ‘R/K/H’ side-chain, these residues form a salt bridge (Kumar and Nussinov, 2002). Additionally, D/E at the 101st position complements this interaction by forming hydrogen bonds or salt bridges with antigen surface residues, where they significantly contribute to the ability of the antibody to recognize and tightly bind to its target, influencing its specificity and affinity (North et al., 2011).

Intriguingly, within our dataset, various combinations of acidic and basic residues existed beyond the 94th R–101st D pairing, as well as instances where no salt bridge-forming residues were present at all, which contradicts the previously reported conserved pattern of the 94th R–101st D salt bridge (Tables S8 and S9). An interesting contrast also emerged in V_H_Hs, as most of them lacked this canonical salt bridge interaction (Table S9). While 63.1% of V_H_s had one of these salt bridges connecting the residues at the 94th and 101st positions (Table S8), V_H_Hs only had a 2.7% incidence (Table S9). Specifically, in 56% of V_H_s, the ‘R-D’ pairing was observed, which results in the strongest interaction, leading to the shortest interatomic distance compared to other combinations of salt bridge-forming residues (Musafia et al., 1995). Therefore, the residues at the 94th and 101st positions are likely to be of importance in humanization/ camelization studies.

A distinctive conformation is achieved through the interaction between the basic 94th residue’s hydrophobic part and the HCDR1, along with a hydrogen bond linking the conserved 103rd ‘W’ to the preceding residue at the 101st position. When a negatively charged residue is at the 101st position without a corresponding positively charged residue at the 94th position, the HCDR3 torso takes on a nonbulged form. In this scenario, the acidic side chain, lacking a salt bridge partner, forms a hydrogen bond with the side chain of the conserved 103rd ‘W,’ creating a notable kink in the structure (Morea et al., 1997). The absence of a charged amino acid at the 94th position of camel V_H_Hs was reported before (Muyldermans et al., 1994). When no salt bridge was observed between the 94th and 101st positions, due to the absence of the 101st residue, the presence of the 103rd ‘W’ residue was not always consistent to confer a bulged kink for favorable additional interactions. Overall, 37% of the V_H_s did not harbor any salt bridge at this position, while this value reached up to 97% in V_H_Hs. This highlights the structural diversity and adaptability of different antibody structures. In light of the diverse structural manifestations observed, particularly regarding the intricate interplay between residues at the 94th, 101st, and 103rd positions, future prospective studies should delve into the functional implications arising from variations in these key structural elements.

### 3.6. Statistical analysis of physicochemical properties of FW residues of V_H_s and V_H_Hs

Next, our analysis extended to the distribution of physicochemical amino acid categories within the FW regions of V_H_s and V_H_Hs. The primary objective was to identify specific physicochemical characteristics that may contribute to the distinctions between these two antibody classes, potentially influencing the folding, stability, or affinity of their functional structures. To accomplish this, we employed an amino acid classification system that encompassed a total of nine distinct categories, as outlined in Table S2, based on EMBOSS Pepstats. The percentage of residues in each sequence for each category was calculated using the EMBOSS Pepstats online tool (Supplementary File 2). It is worth noting that certain amino acids were categorized in multiple subclasses due to their overlapping physicochemical characteristics. As part of our analysis, in addition to the physicochemical properties, we also evaluated the molecular charge and hydropathy (Table S3) properties of the V_H_s and V_H_Hs.

We employed both a logistic regression model and a machine learning approach to evaluate and analyze these comparisons. The logistic regression modeling process was performed for each FW region (FW1–4) collectively (Table; Table S10) and individually (Table S11). For this analysis, we specifically focused on the FW regions, as the hypervariability of CDRs created many confounding factors, making it difficult to ensure that the results were due to the differences between the antibody formats. When we applied our chosen statistical model with all physicochemical properties, the contribution of the variables could potentially affect the overall results, leading to otherwise significant findings being ruled out due to multicollinearity. Furthermore, the outcomes of the analysis were overly intricate, making it challenging to grasp the implications.

The collective statistical analysis provided valuable insights into the outcomes of the logistic regression, primarily focusing on the Beta and Exp(Beta) values (Table). Exp(Beta) values higher than 1 indicate that the physicochemical characteristics were overrepresented in V_H_Hs (e.g., FW1-tiny and aromatic), while lower values indicate the reverse (e.g., FW1-small and basic). The most significant differences occurred in tiny/small and/ or hydrophobic/hydrophilic contents in almost all FW regions. The statistical significance of the collective model is further detailed in Table S10.

We also conducted logistic regression analysis of individual FWs, with the outputs being evaluated based on the previously mentioned scoring criteria (Table S11). Most of the results confirmed the whole V_H_ sequence model. Upon performing the individual analysis, distinct characteristics were observed for each FW region. In addition to the previous findings, FW1 residues were more hydrophilic. FW3 displayed specifically hydrophilic residues compared to the nonhydrophobic residues identified in the V_H_-wide analysis. Notably, FW4 was characterized as hydrophilic and was found to be the shortest among all other framework regions. It also had a very low constant value, leading to lower variable importance (VI). This feature in the FW4 structure underscored its specific role, emphasizing the significance of individual analysis in capturing nuanced details that might be overlooked in collective assessments.

To further validate the accuracy of our feature selection, we also used the Boruta Algorithm-Random Forest. The Boruta algorithm, as a wrapper around the random forest algorithm, played a pivotal role in highlighting the physicochemical variables with the highest VI scores. Combining the Boruta algorithm with logistic regression modeling yielded compelling results in our study. This dual approach involved the integration of a statistical method and a machine learning technique, providing a comprehensive and reliable perspective on the analyzed data, as well as validating our findings through established statistical techniques, ensuring the credibility and accuracy of our study outcomes. We ranked the VI scores for each modeled feature (Figure S3) and the frequency of amino acids in each region (HCDRs and FWs) (Figure S4). The results indicated the accuracy of the logistic regression analysis model, as features with very high and very low Exp(Beta) scores, such as hydrophilic FW4 residues and tiny FW1 residues, were ranked highly compared to other features. It is important to note that while the hydrophilic residues of FW2 are well known, other features, such as the tiny residues in FW1 and the small residues of FW3, were ranked higher in comparison, both supported by our motif conservation analysis. This highlights the importance of FW1 and FW3, which might contain elements of higher impact for the structure of the V_H_ domain than the hydrophilic FW2 residues. When examining the individual amino acid contributions in different regions, we noted that the residues found among the hallmark residues of FW2, especially ‘FW2_Leu’ and ‘FW2_Phe,’ were ranked near the top of all amino acids, both of which contribute to the more hydrophilic interface of V_H_Hs. Similarly, the high ‘G’ content of FW1 likely contributes to the importance of tiny residues in the FW1 of V_H_Hs. Finally, the highly conserved ‘S’ residues in FW3 in V_H_Hs are often substituted for other residues in V_H_s, which may explain its high ranking.

FW4 was found to be the shortest among all other framework regions and it had a very low constant value, leading to lower VI scores. This might indicate that FW4 is not of high importance for humanization/camelization approaches. Alternatively, the short length of FW4 might mask the importance of individual features when comparing V_H_s and V_H_Hs, due to the small range of each feature.

### 3.7. Physicochemical characteristics of individual residues in V_H_s and V_H_Hs

Finally, we examined the physicochemical properties of V_H_s and V_H_Hs on the residue level (Figure S5). The results showed that several residues have higher variance in certain physicochemical characteristics between V_H_s and V_H_Hs compared to the overall sequence. In FW1, residues with similar physicochemical characteristic differences included the 6th and 6Ath positions, as well as the 10th and 23rd positions. In addition to the hallmark residues, where each residue displayed the appropriate physiochemical characteristics, the 35th, 40th, and 50th positions of FW2 also showed high differences, with V_H_s favoring aromatic and hydrophobic residues while V_H_Hs showed a higher degree of smaller and nonpolar residues at the same positions. FW3 showed similar residue traits in the 60th, 62nd, and 94th positions. Interestingly, FW4 was entirely devoid of such residues, with the 108th residue instead favoring small, nonpolar, hydrophilic residues in V_H_s and hydrophobic residues in V_H_Hs.

One feature of interest among the physicochemical characteristics was the distribution of hydrophobic and hydrophilic amino acids among the FWs of V_H_s and V_H_Hs, respectively. While the absence of the V_H_–V_L_ promotes a higher degree of hydrophilic residues in V_H_Hs, these residues assist in the proper folding and packing of the domain into its appropriate shape. Other than the 27th position, which strongly favored aromatic, nonpolar residues and slightly favored hydrophilic residues in V_H_s, very few nonalternating residues had strong differences between V_H_s and V_H_Hs. This confirmed our previous statement that the major differences in HCDR regions were caused by target recognition rather than species of origin. However, there was a weak preference across multiple residues for less polar and more hydrophilic residues in HCDR3s compared to those of V_H_Hs. The absence of such differences in HCDR3 otherwise further points to its importance in epitope recognition.

This study has revealed significant distinctions between antibodies and nanobodies, emphasizing the longer and less variable HCDR3s, specific binding-enhancing residues (at the 50th and 94th positions in antibodies and the 29th and 51st positions in nanobodies), and distinct motif conservation patterns in the latter. Analyzing the antigen binding scores allows for the identification of crucial residue locations on CDRs, providing valuable insights for studies on CDR grafting. Nanobodies exhibit a higher prevalence of species-specific noncanonical disulfide bonds. Additionally, a prominent salt bridge concentration was highlighted between the 94th and 101st positions in the antibodies, which is missing in the nanobodies. Unique physicochemical characteristics of nanobodies’ FWs were revealed compared to IgGs: the residues in FW1 were predominantly tiny, hydrophilic, and noncharged; those in FW2 featured a smaller size and higher hydrophilicity; the FW3 region’s residues were small, nonpolar, and nonhydrophobic; and FW4 residues exhibited small, nonaromatic, and polar traits. The logistic regression-based model employed feature selection based on p-value scores and information criteria. In contrast, the Boruta algorithm, a decision tree-based method, randomly explores features using Monte Carlo techniques (Sanchez-Pinto et al., 2018). Logistic regression follows a minimal optimal approach, relying on a small subset of features that minimize error on a chosen classifier. On the other hand, the Boruta algorithm, by virtue of its random exploration, can discover solutions closer to the global optimum. This characteristic provides the Boruta algorithm with a significant advantage in effectively handling interactions between physicochemical variables. The insights of this study are pivotal for converting conventional antibodies into single-domain V_H_ formats and providing valuable considerations for camelization processes. These findings contribute to the advancement of antibody engineering strategies, drawing inspiration from natural evolution.

It is important to consider potential limitations and sources of error in the data analysis of this study, such as partial missing sequence information. In some cases, the sample sequences may have unresolved residues due to limitations in experimental techniques like crystallography. These missing residues were more commonly found within the constant regions of the antibodies, which are generally well conserved in both sequence and structure. The total percentage of nonresolved residues was within acceptable levels and did not have any major impact on our results. Additionally, the accuracy of structural predictions for noncanonical bonds may introduce a margin of error.

This study has enhanced our knowledge of antibody engineering and provides a foundation for the rational design and development of novel antibody formats with improved characteristics and functionalities. By studying the physicochemical characteristics and structural adaptations of V_H_Hs compared to conventional V_H_s, we gain insights into how nature has adapted antibody formats to optimize their function in different contexts. In particular, our findings contribute to the understanding of engineering approaches for the efficient conversion of full-length IgG into smaller V_H_ format through camelization and assist in the mitigation of immunogenicity concerns, inspired by nature’s ingenuity.

## Supplementary Files

### 1. Supplementary File 1: Statistical Methods

#### 1.1. Binary logistic regression model

As the strategy for selecting important physicochemical predictors, stepwise regression backward elimination with the likelihood ratio test was used.

The binary logistic regression model is shown in Eq. (1).


(1) $$\text{ln}\;(\frac{p}{1-p})=\beta_{0}+\beta_{1}.X_{1}+\beta_{2}.X_{2}+\cdots +\beta_{k}.X_{k}$$

Here, p stands for the probability of the presence of V_H_Hs.

The binary logistic regression model relied on the assumption of independence (multicollinearity) among predictors, along with linearity. To control the multicollinearity for each predictor in the model, the variance inflation factor (VIF) was calculated. Removing predictors with high VIF values (>10) helped to reduce the multicollinearity and improve the accuracy of the binary logistic regression model. This model, refined through stepwise elimination, provides a succinct representation of the influential factors in the classification process, thereby offering a robust foundation for understanding the underlying physicochemical determinants governing the distinctions between VHs and V_H_Hs. By implementing this VIF-based predictor selection strategy, we ensured a more robust and reliable assessment of the relationship between VHs and V_H_Hs.

### 1.2. Boruta Algorithm

The Boruta algorithm is a wrapper built around the Random Forest (RF) classification algorithm. It tries to capture all the important, interesting features in a dataset with respect to an outcome variable. The Boruta algorithm was fitted jointly to data for amino acids for all regions of VHs and V_H_Hs. The responses were ordered from left to right by their importance for classification. The importance measure was defined as the Z-score of the mean decrease in accuracy (normalized permutation importance). Operating as an ensemble learning method, the Boruta algorithm was used to capture all relevant and impactful features with respect to the outcome variable, which, in this case, pertained to the classification of VHs and V_H_Hs.

### 1.3. Statistical significance calculations

McFadden’s R^2^ or McFadden’s pseudo-R^2^ is a measure of the goodness of fit of the binary logistic regression model. It measures how well the model predicts the outcome. The area under the curve (AUC) is an overall summary of the diagnostic accuracy of the binary logistic regression model. If the AUC is greater than 0.5, the model performs well at classifying antibody sequences as VHs or V_H_Hs.

McFadden’s R^2^ assesses the effectiveness of the model in predicting the outcome, offering insights into the proportion of variance explained by the predictors.

The AUC is particularly useful in assessing the model’s ability to correctly classify antibody sequences as VHs and V_H_Hs.

## 2. Supplementary File 2: Tables and Figures

Supplementary data available at: https://data.mendeley.com/datasets/4g5njh2ct2/1

**Table S1:** Definition of constant and variable regions in VH and V_H_H sequences based on the Chothia numbering scheme (FW1–4: framework regions, HCDR1–3: complementarity determining regions).

VH/V _H_H region	Chothia residue
FW1	1–25
HCDR1	26–32
FW2	33–51
HCDR2	52–56
FW3	57–94
HCDR3	95–102
FW4	103–113

**Table S2:** Classification of physicochemical properties shown as single-letter amino acid codes defined by EMBOSS Pepstats.

Property	Amino acid residues
Tiny	A+C+G+S+T
Small	A+B+C+D+G+N+P+S+T+V
Aliphatic	A+I+L+V
Aromatic	F+H+W+Y
Non-polar	A+C+F+G+I+L+M+P+V+W+Y
Polar	D+E+H+K+N+Q+R+S+T+Z
Charged	B+D+E+H+K+R+Z
Basic	H+K+R
Acidic	B+D+E+Z

**Table S3:** Amino acid hydropathy classes in single-letter amino acid codes.

Property	Amino acid residues
Hydrophilic	D+E+K+N+R+Q
Neutral	G+H+P+T+S+Y
Hydrophobic	A+C+F+I+L+M+V+W

**Table S4:** Average length of HCDRs in VHs categorized by species, based on our dataset.

Species	HCDR1 length	HCDR2 length	HCDR3 length
*Cricetulus migratorius*	7	6	7.9
*Homo sapiens*	7	6.4	10.5
*Homo sapiens* **/** *Mus musculus*	7.4	5.8	9.9
*Mus musculus*	7.1	6	9.8
*Mus musculus* ***/*** *Homo sapiens*	7.1	5.7	10.1
*Pan troglodytes*	5	7	15
*Rattus norvegicus*	7	5.7	8.2
*Rattus norvegicus* **/** *Homo sapiens*	7	6	8
Average VHs length	7.2	5.9	11.9

**Table S5:** Average length of HCDRs in V_H_Hs categorized by species, based on our dataset.

Species	HCDR1 length	HCDR2 length	HCDR3 length
Camelidae	7	5.8	14.5
*Camelus bactrianus*	7.8	6.1	15.6
*Camelus dromedarius*	6.9	5.7	13.2
*Lama glama*	6.9	5.8	13.9
*Vicugna pacos*	7.1	5.8	12.5
Average V _H_Hs length	6.9	5.8	13.5

**Table S6:** Total number of canonical and noncanonical disulfide bridges in VHs for each species from our dataset.

Species	No bond	Canonical bonds	Noncanonical bonds	Total VHs
*Cricetulus migratorius*	-	10	-	10
*Homo sapiens*	1	2456	130	2457
*Homo sapiens* **/** *Mus musculus*	-	26	-	26
*Mus musculus*	3	2287	2	2290
*Mus musculus* **/** *Homo sapiens*	-	188	-	188
*Pan troglodytes*	-	4	-	4
*Rattus norvegicus*	-	53	-	53
*Rattus norvegicus* **/** *Homo sapiens*	-	2	-	2
Total	4	5026	132	5030

**Table S7:** Total number of canonical and noncanonical disulfide bridges in V_H_Hs for each species from our dataset.

Species	No bond	Canonical bonds	Noncanonical bonds	Total V _H_Hs
Camelidae	-	65	3	65
*Camelus bactrianus*	-	63	4	63
*Camelus dromedarius*	-	263	26	263
*Lama glama*	1	669	16	670
*Vicugna pacos*	-	199	20	199
Total	1	1259	69	1260

**Table S8:** Total number of canonical (R94–D101) and noncanonical salt bridges (combinations of R/H/K 94–D/E101 excluding canonical ones) in VHs on species basis.

Species	Canonical salt bridges	Noncanonical salt bridges	No salt bridge	Total VHs
*Cricetulus migratorius*	5	-	5	10
*Homo sapiens*	1409	282	766	2457
*Homo sapiens* **/** *Mus musculus*	10	-	16	26
*Mus musculus*	1291	44	955	2290
*Mus musculus* **/** *Homo sapiens*	80	5	103	188
*Pan troglodytes*	4	-	-	4
*Rattus norvegicus*	32	10	11	53
*Rattus norvegicus* **/** *Homo sapiens*	-	2	-	2
Total	2831	343	1856	5030

**Table S9:** Total number of canonical (R94–D101) and noncanonical salt bridges (combinations of R/H/K 94–D/E101 excluding canonical ones) in V_H_Hs on species basis.

Species	Canonical salt bridges	Noncanonical salt bridges	No salt bridge	Total V _H_Hs
Camelidae	-	-	65	65
*Camelus bactrianus*	-	1	62	63
*Camelus dromedarius*	2	-	261	263
*Lama glama*	12	13	645	670
*Vicugna pacos*	5	2	192	199
Total	19	16	1225	1260

**Table S10:** Logistic regression analysis results incorporating all four FW regions, FW1–4, collectively along with the various physicochemical properties.

FWs	Physicochemical property	Variable importance	VIF *	McFadden R ^2^	AUC
FW1	Tiny	5.628	3.453	0.9730	0.999
	Small	3.306	2.891		
	Aliphatic	2.283	5.979		
	Aromatic	2.575	1.315		
	Basic	4.319	3.612		
	Acidic	2.037	2.544		
	Polar	2.336	6.038		
FW2	Tiny	7.400	6.938		
	Aromatic	2.833	3.587		
	Aliphatic	3.624	5.596		
	Polar	2.338	5.049		
	Basic	3.830	2.382		
	Hydrophobic	4.603	7.424		
	Hydrophilic	6.388	8.388		
FW3	Small	6.143	3.103		
	Aromatic	2.249	2.578		
	Polar	2.649	5.999		
	Basic	2.618	1.724		
	Hydrophobic	2.885	6.332		
FW4	Tiny	3.414	1.880		
	Aliphatic	3.785	1.823		
	Aromatic	5.984	3.002		
	Hydrophilic	9.123	4.596		

* The variables not shown in the table were statistically insignificant based on their VIF values.

**Table S11:** Logistic regression analysis incorporating all four FW regions, FW1–4, individually along with various physicochemical properties.

FWs	Physicochemical property	Beta	Exp(Beta)	Variable importance	VIF	McFadden R ^2^	AUC
FW1	Constant	−39.139	0.0000	-	-	0.688	0.97
	Tiny	0.725	2.065	24.964	3.088		
	Aliphatic	−0.065	0.937	1.968	4.237		
	Aromatic	0.643	1.902	11.503	1.423		
	Polar	−0.257	0.773	10.533	4.069		
	Basic	−0.533	0.587	18.738	2.016		
	Acidic	−0.331	0.718	14.686	1.131		
	Hydrophobic	0.059	1.062	2.639	3.806		
	Hydrophilic	0.806	2.238	19.970	6.629		
FW2	Constant	−39.591	0.00	-	-	0.677	0.9539
	Tiny	0.501	1.65	26.48	4.29		
	Aliphatic	−0.139	0.87	7.75	3.65		
	Aromatic	0.202	1.22	9.43	2.34		
	Polar	−0.123	0.89	10.15	2.26		
	Basic	0.182	1.19	10.73	1.44		
	Hydrophobic	0.207	1.23	16.43	3.48		
	Hydrophilic	0.649	1.91	27.39	5.25		
FW3	Constant	15.233	4126747	-	-	0.535	0.9432
	Tiny	0.295	1.343	13.706	5.26		
	Small	0.552	1.736	26.822	2.75		
	Aliphatic	−0.083	0.920	3.307	3.48		
	Aromatic	−0.082	0.921	2.782	1.75		
	Polar	−0.699	0.497	27.249	3.65		
	Hydrophobic	−0.225	0.798	11.716	4.12		
	Hydrophilic	0.318	1.374	12.437	4.21		
FW4	Constant	−1.120	0.326	-	-	0.799	0.9711
	Tiny	−0.093	0.911	8.729	1.090		
	Small	0.129	1.138	5.046	1.861		
	Aromatic	−0.119	0.888	2.582	1.172		
	Acidic	−0.553	0.575	4.116	1.029		
	Hydrophobic	−0.110	0.895	8.000	1.887		
	Hydrophilic	0.656	1.927	32.363	1.097		

Figure S1 Graphical logo representation of multiple sequences of the complete VH dataset based on species. A) *Cricetulus migratorius*, B) *Homo sapiens*, C) *Homo sapiens*/*Mus musculus*, D) *Mus musculus*, E) *Mus musculus*/*Homo sapiens*, F) *Pan troglodytes*, and G) *Rattus norvegicus*. Multiple sequences were aligned based on the Chothia numbering scheme. The relative frequency of each amino acid position is quantified by logo size in terms of bits. Color codes are based on amino acid biochemical properties: basic: K, R (purple); acidic: D, E (green); aliphatic: A, I, L, V (blue); aromatic: F, H, W, Y (orange); Cys bonds: C (yellow); mostly small: G, M, N, P, Q, S, T (dark gray). Gray boxes show constant FW regions and dark blue boxes show variable HCDR zones.

Figure S2 Graphical representation of multiple sequences of the V_H_H datasets by logos based on species. A) Camelidae, B) *Camelus bactrianus*, C) *Camelus dromedarius*, D) *Lama glama*, and E) *Vicugna pacos*. Multiple sequences were aligned based on the Chothia numbering scheme. The relative frequency of each amino acid position was quantified by logo size in terms of bits. The color codes were based on amino acid biochemical properties: basic: K, R (purple); acidic: D, E (green); aliphatic: A, I, L, V (blue); aromatic: F, H, W, Y (orange); Cys bonds: C (yellow); mostly small: G, M, N, P, Q, S, T (dark gray). Gray boxes show constant FW regions and dark blue boxes show variable HCDR zones.

Figure S3 A box-and-whiskers plot showing the Boruta Algorithm-Random Forest ranking of the variable importance scores of features used for logistic regression modeling analysis.

Figure S4 A box-and-whiskers plot showing the Boruta Algorithm-Random Forest ranking of the variable importance scores of each amino acid–region association. Blue boxes correspond to the minimal, average, and maximum Z-scores of shadow features in Figure S3. Red boxes indicate variables not contributing significantly to accurate classification. Green boxes indicate the amino acids contributing significantly to the classification that were selected for further evaluation. The centers of the boxes correspond to the median. The lower and upper hinges of the boxes correspond to the first and third quartiles of the data. The upper and lower whiskers extend from the hinges to the largest and smallest values, respectively, no further than 1.5 times the interquartile range from the hinges. Data beyond the end of the whiskers were plotted individually.

Figure S5 Heatmaps display the differences in physicochemical characteristics of residues between VHs and V_H_Hs. Rows represent individual residues, while columns indicate physicochemical properties of interest as determined in logistic regression analysis. Cell color indicates which antibody format contains a higher percentage of the indicated property in the residue. Red cells indicate a higher property in V_H_Hs, while blue cells indicate a higher property in VHs. Rows in gray indicate alternating positions, where either VHs or V_H_Hs lack the corresponding value, preventing a difference from being calculated. Individual heatmaps show (A) FW1, (B) HCDR1, (C) FW2, (D) HCDR2, (E) FW3, (F) HCDR3, and (G) FW4 residues. The color scale was set to 50 to –50, with higher or lower difference values being set to the set maximum or minimum, respectively.

## Figures and Tables

**Table:** Logistic regression analysis results based on EMBOSS Pepstats output incorporating all FW1–4 regions, along with the various physicochemical properties.

		Logistic Regression			Confidence interval	
FWs	Physicochemical property	Beta	Exp(Beta)	p-values	Lower bound	Upper bound
	Constant	−47.781	0.000	1.014.e^−04^		
FW1	Tiny	0.517	1.678	1.823.e^−08^	1.4205	2.1739
	Small	−0.312	0.732	9.455.e^−04^	0.5942	0.8749
	Aliphatic	0.206	1.228	2.241.e^−02^	0.9629	1.4402
	Aromatic	0.745	2.107	1.002.e^−02^	1.2448	4.0962
	Basic	−0.366	0.693	1.569.e^−05^	0.5717	0.8254
	Acidic	0.162	1.176	4.168.e^−02^	1.0103	1.4292
	Polar	0.146	1.157	1.956.e^−02^	1.0096	1.2873
FW2	Tiny	0.635	1.887	1.358.e^−13^	1.5956	2.4403
	Aromatic	0.247	1.279	4.606.e^−03^	1.0703	1.5909
	Aliphatic	−0.328	0.720	2.898.e^−04^	0.5298	0.8343
	Polar	−0.150	0.861	1.939.e^−02^	0.7367	0.9975
	Basic	0.330	1.392	1.279.e^−04^	1.1885	1.8180
	Hydrophobic	0.328	1.388	4.172.e^−06^	1.2448	1.8178
	Hydrophilic	0.647	1.909	1.676.e^−10^	1.5592	2.4595
FW3	Small	0.578	1.782	8.100.e^−10^	1.5255	2.7124
	Aromatic	−0.360	0.698	2.448.e^−02^	0.4978	0.9796
	Polar	−0.311	0.733	8.081.e^−03^	0.5951	0.9799
	Basic	−0.342	0.710	8.842.e^−03^	0.5525	0.9388
	Hydrophobic	−0.265	0.767	3.919.e^−03^	0.5376	0.9429
FW4	Tiny	−0.153	0.858	6.413.e^−04^	0.7049	0.9999
	Aliphatic	−0.170	0.844	1.539.e^−04^	0.6246	0.9680
	Aromatic	−0.577	0.562	2.182.e^−09^	0.4168	0.6628
	Hydrophilic	0.867	2.380	7.299.e^−20^	1.8508	3.069
