# Supplementary figures and images for: Physicochemical differences between camelid single-domain antibodies and mammalian antibodies
Source: Turk J Biol. 2023 Dec 7;47(6):423–36. doi: 10.55730/1300-0152.2676 (PMC11045209; doi:10.55730/1300-0152.2676)

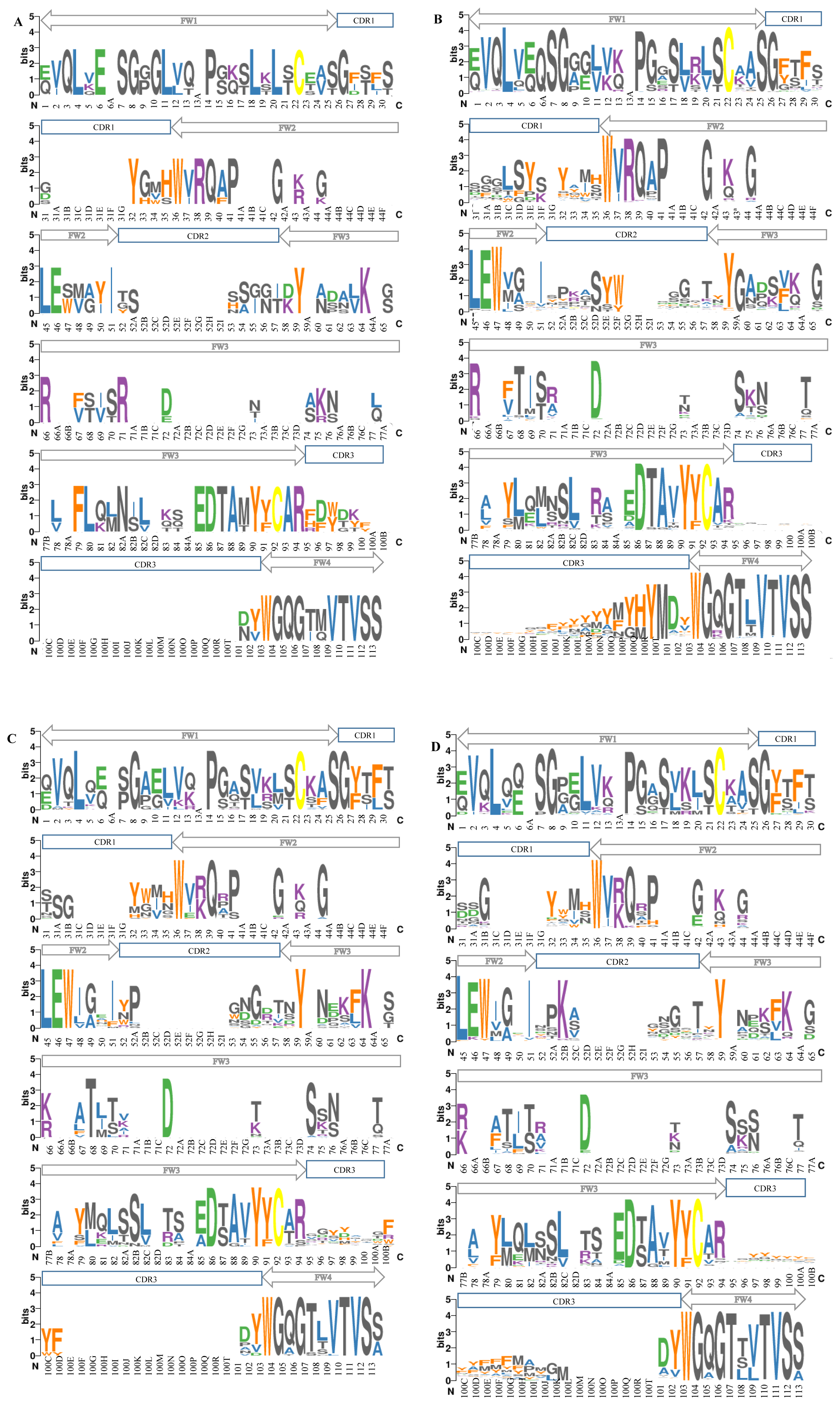

Supplement: Figure S1 — Graphical logo representation of multiple sequences of the complete VH dataset based on species. A) Cricetulus migratorius, B) Homo sapiens, C) Homo sapiens/Mus musculus, D) Mus musculus, E) Mus musculus/Homo sapiens, F) Pan troglodytes, and G) Rattus norvegicus. Multiple sequences were aligned based on the Chothia numbering scheme. The relative frequency of each amino acid position is quantified by logo size in terms of bits. Color codes are based on amino acid biochemical properties: basic: K, R (purple); acidic: D, E (green); aliphatic: A, I, L, V (blue); aromatic: F, H, W, Y (orange); Cys bonds: C (yellow); mostly small: G, M, N, P, Q, S, T (dark gray). Gray boxes show constant FW regions and dark blue boxes show variable HCDR zones. [file tjb-47-06-423s1a.tif]

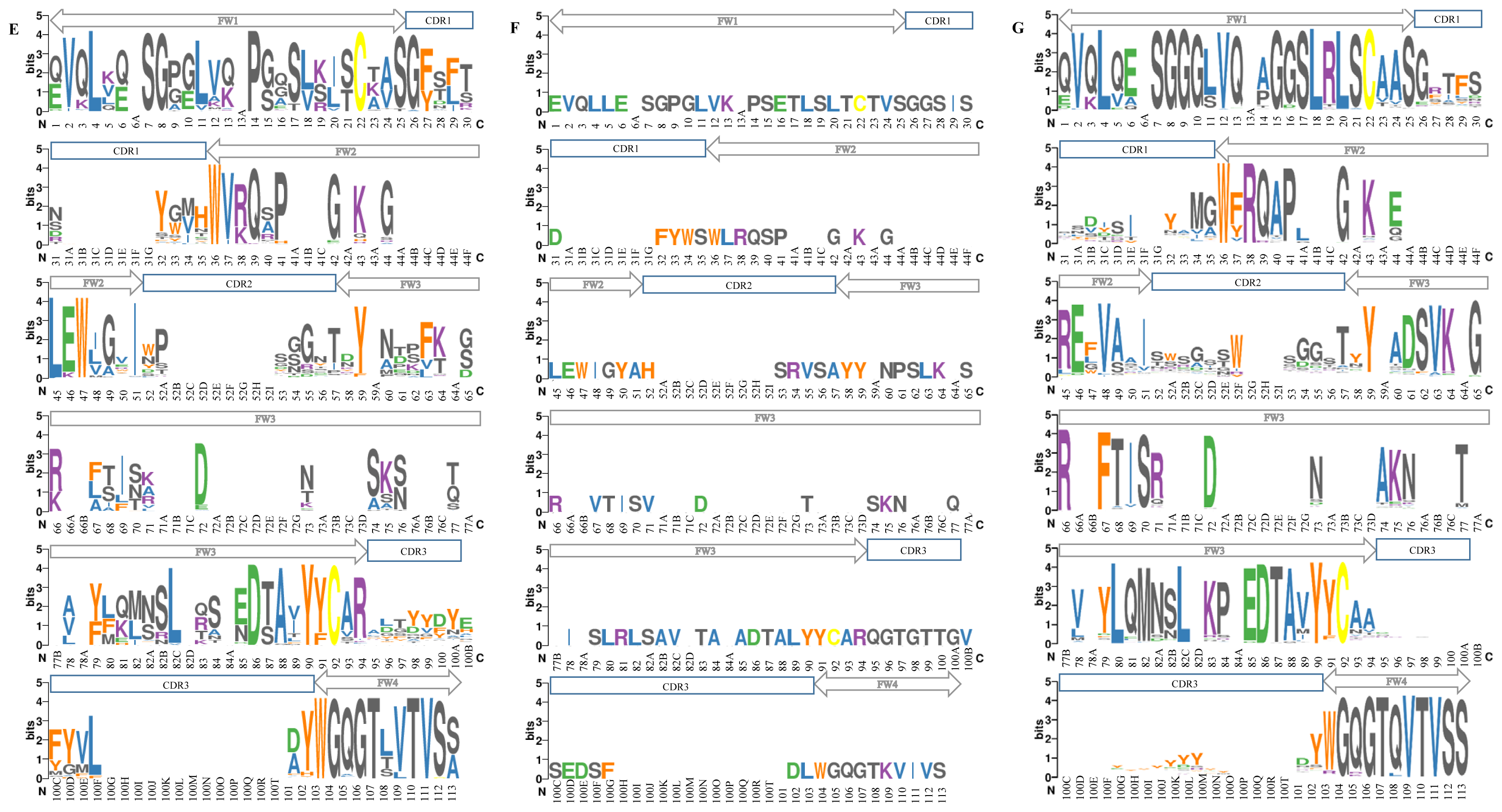

Supplement: Figure S1 — Graphical logo representation of multiple sequences of the complete VH dataset based on species. A) Cricetulus migratorius, B) Homo sapiens, C) Homo sapiens/Mus musculus, D) Mus musculus, E) Mus musculus/Homo sapiens, F) Pan troglodytes, and G) Rattus norvegicus. Multiple sequences were aligned based on the Chothia numbering scheme. The relative frequency of each amino acid position is quantified by logo size in terms of bits. Color codes are based on amino acid biochemical properties: basic: K, R (purple); acidic: D, E (green); aliphatic: A, I, L, V (blue); aromatic: F, H, W, Y (orange); Cys bonds: C (yellow); mostly small: G, M, N, P, Q, S, T (dark gray). Gray boxes show constant FW regions and dark blue boxes show variable HCDR zones. [file tjb-47-06-423s1b.tif]

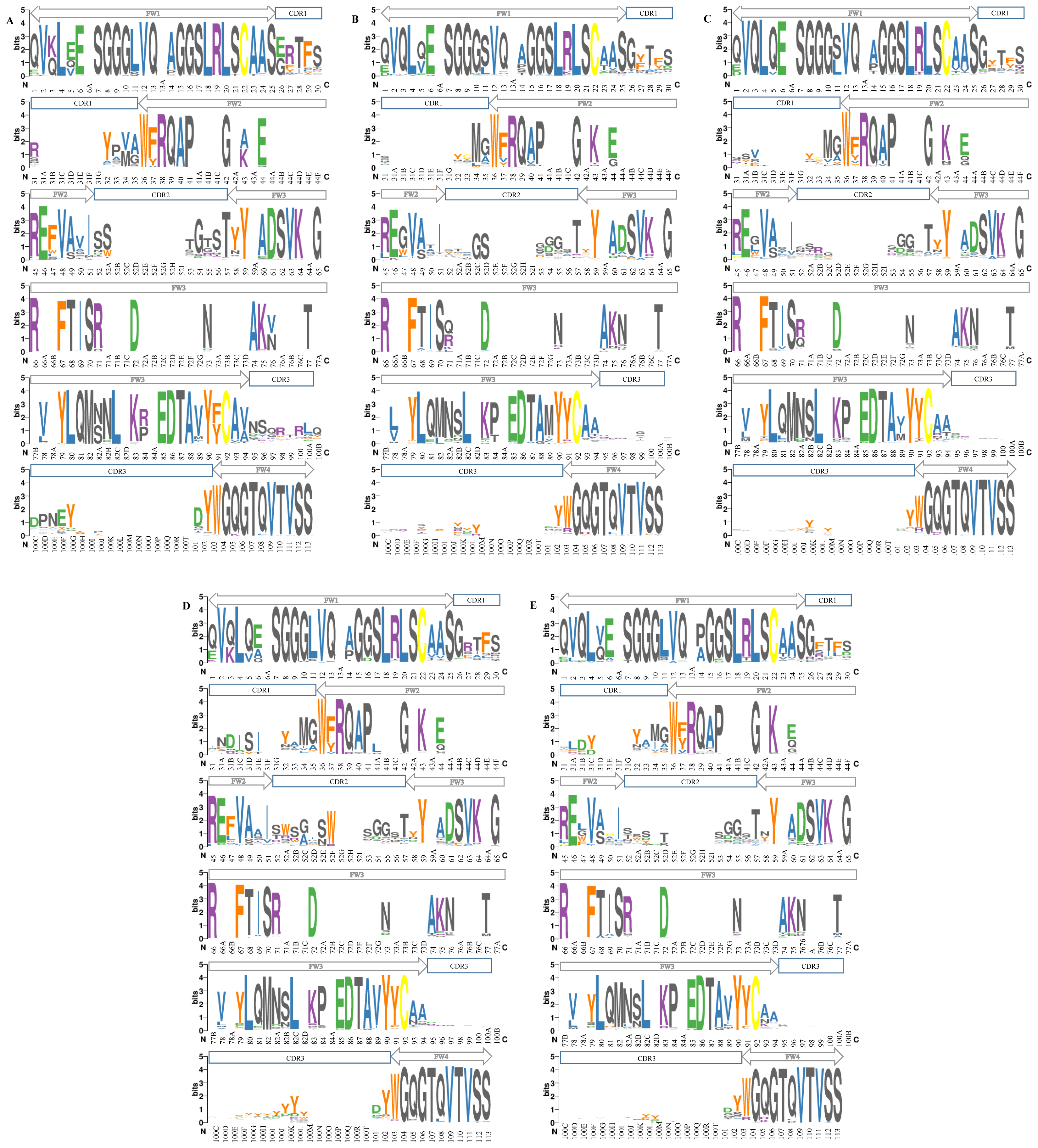

Supplement: Figure S2 — Graphical representation of multiple sequences of the VHH datasets by logos based on species. A) Camelidae, B) Camelus bactrianus, C) Camelus dromedarius, D) Lama glama, and E) Vicugna pacos. Multiple sequences were aligned based on the Chothia numbering scheme. The relative frequency of each amino acid position was quantified by logo size in terms of bits. The color codes were based on amino acid biochemical properties: basic: K, R (purple); acidic: D, E (green); aliphatic: A, I, L, V (blue); aromatic: F, H, W, Y (orange); Cys bonds: C (yellow); mostly small: G, M, N, P, Q, S, T (dark gray). Gray boxes show constant FW regions and dark blue boxes show variable HCDR zones. [file tjb-47-06-423s2.tif]

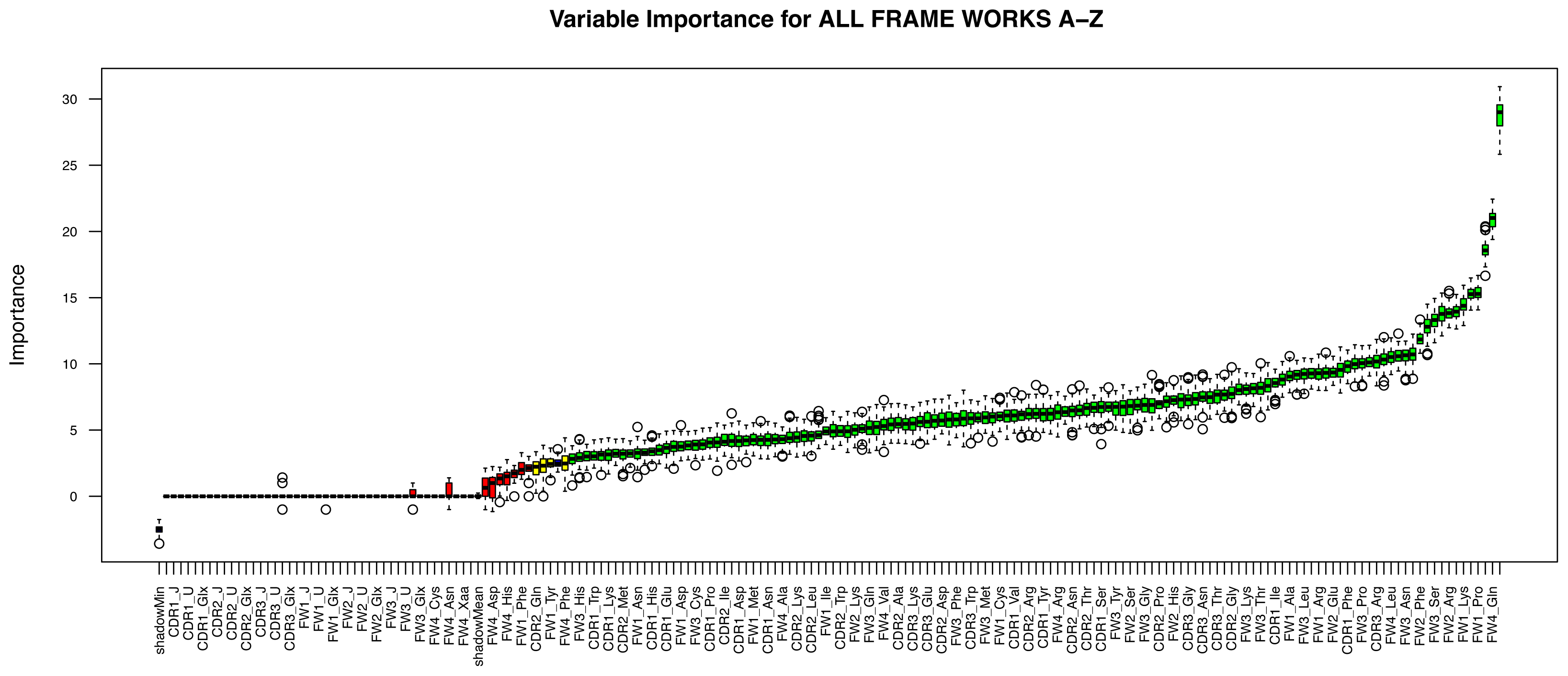

Supplement: Figure S3 — A box-and-whiskers plot showing the Boruta Algorithm-Random Forest ranking of the variable importance scores of features used for logistic regression modeling analysis. [file tjb-47-06-423s3.tif]

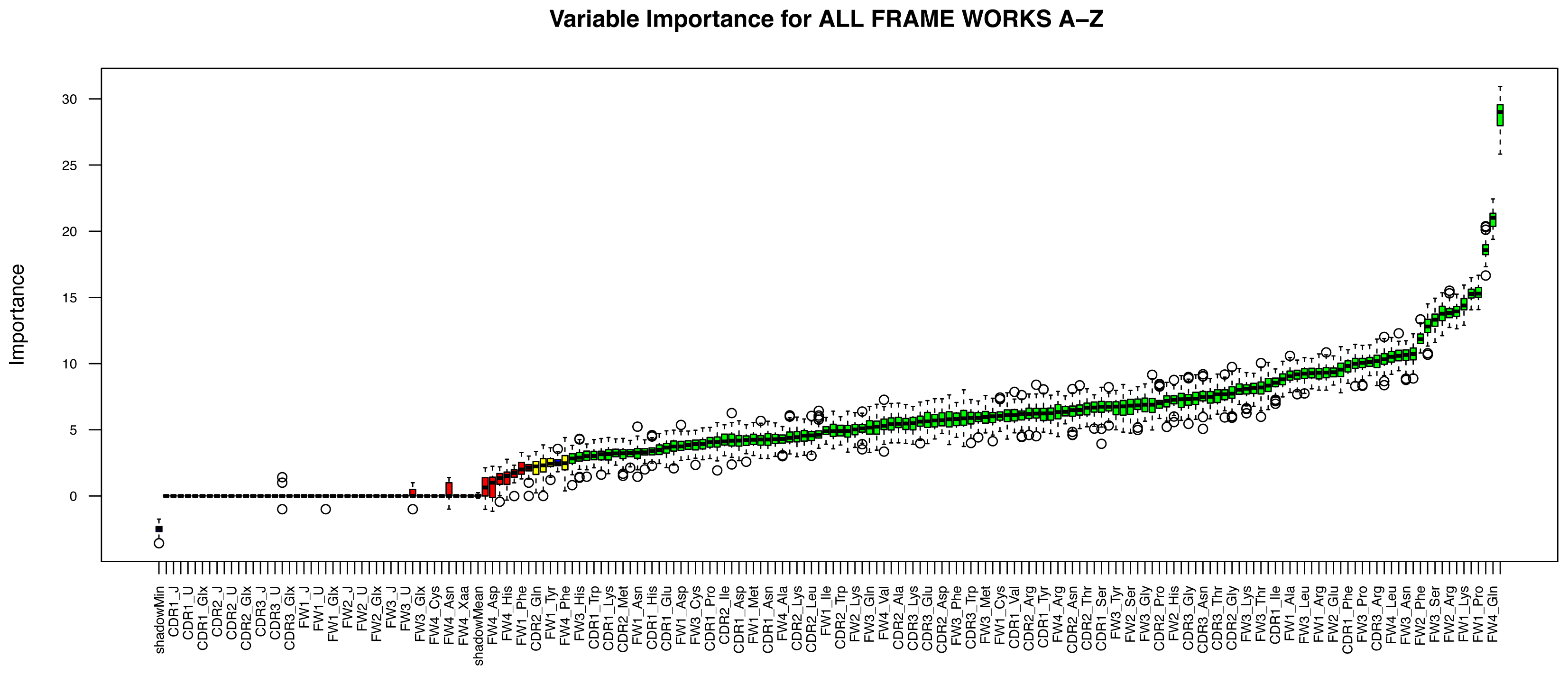

Supplement: Figure S4 — A box-and-whiskers plot showing the Boruta Algorithm-Random Forest ranking of the variable importance scores of each amino acid–region association. Blue boxes correspond to the minimal, average, and maximum Z-scores of shadow features in Figure S3. Red boxes indicate variables not contributing significantly to accurate classification. Green boxes indicate the amino acids contributing significantly to the classification that were selected for further evaluation. The centers of the boxes correspond to the median. The lower and upper hinges of the boxes correspond to the first and third quartiles of the data. The upper and lower whiskers extend from the hinges to the largest and smallest values, respectively, no further than 1.5 times the interquartile range from the hinges. Data beyond the end of the whiskers were plotted individually. [file tjb-47-06-423s4.tif]

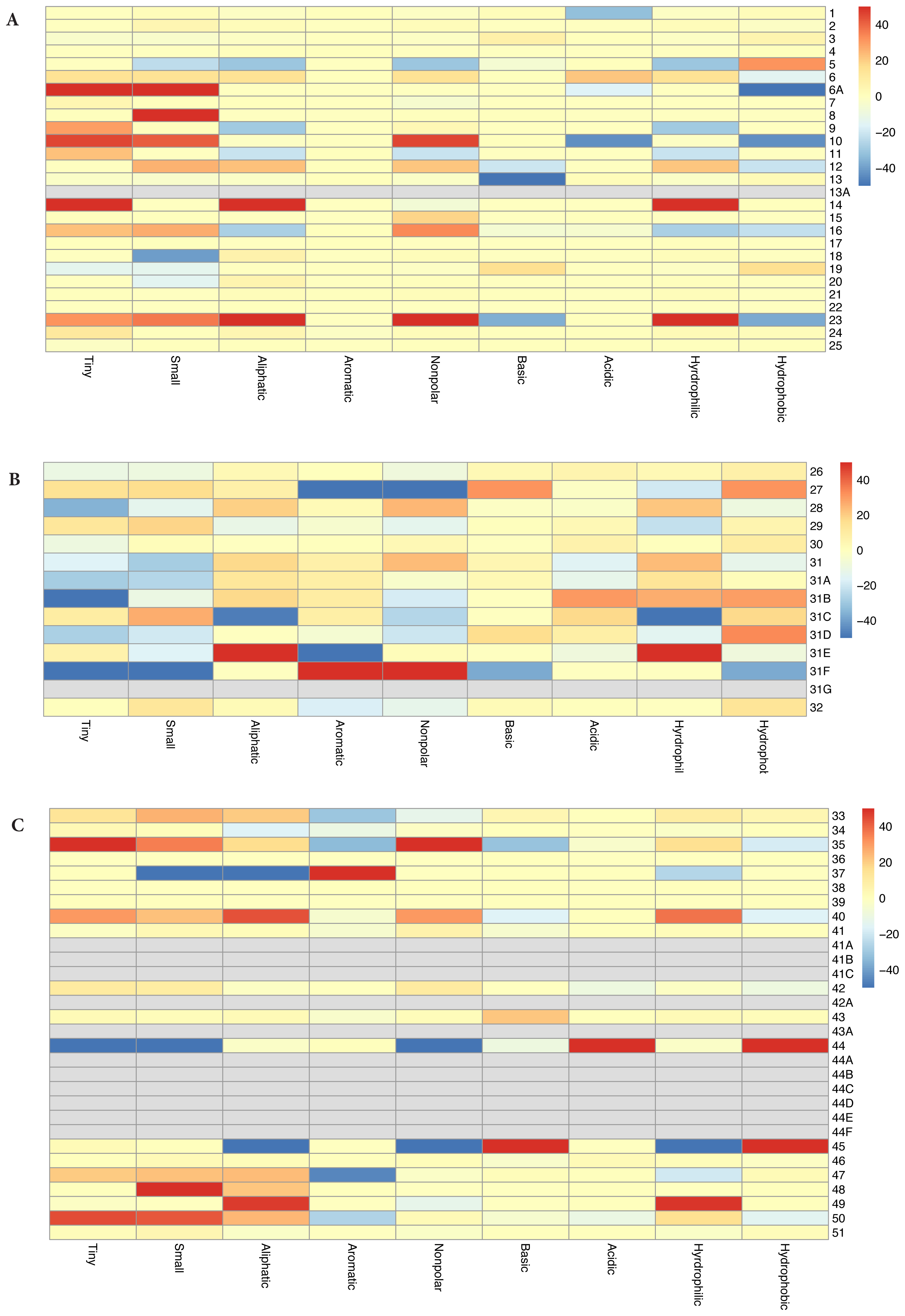

Supplement: Figure S5 — Heatmaps display the differences in physicochemical characteristics of residues between VHs and VHHs. Rows represent individual residues, while columns indicate physicochemical properties of interest as determined in logistic regression analysis. Cell color indicates which antibody format contains a higher percentage of the indicated property in the residue. Red cells indicate a higher property in VHHs, while blue cells indicate a higher property in VHs. Rows in gray indicate alternating positions, where either VHs or VHHs lack the corresponding value, preventing a difference from being calculated. Individual heatmaps show (A) FW1, (B) HCDR1, (C) FW2, (D) HCDR2, (E) FW3, (F) HCDR3, and (G) FW4 residues. The color scale was set to 50 to –50, with higher or lower difference values being set to the set maximum or minimum, respectively. [file tjb-47-06-423s5a.tif]

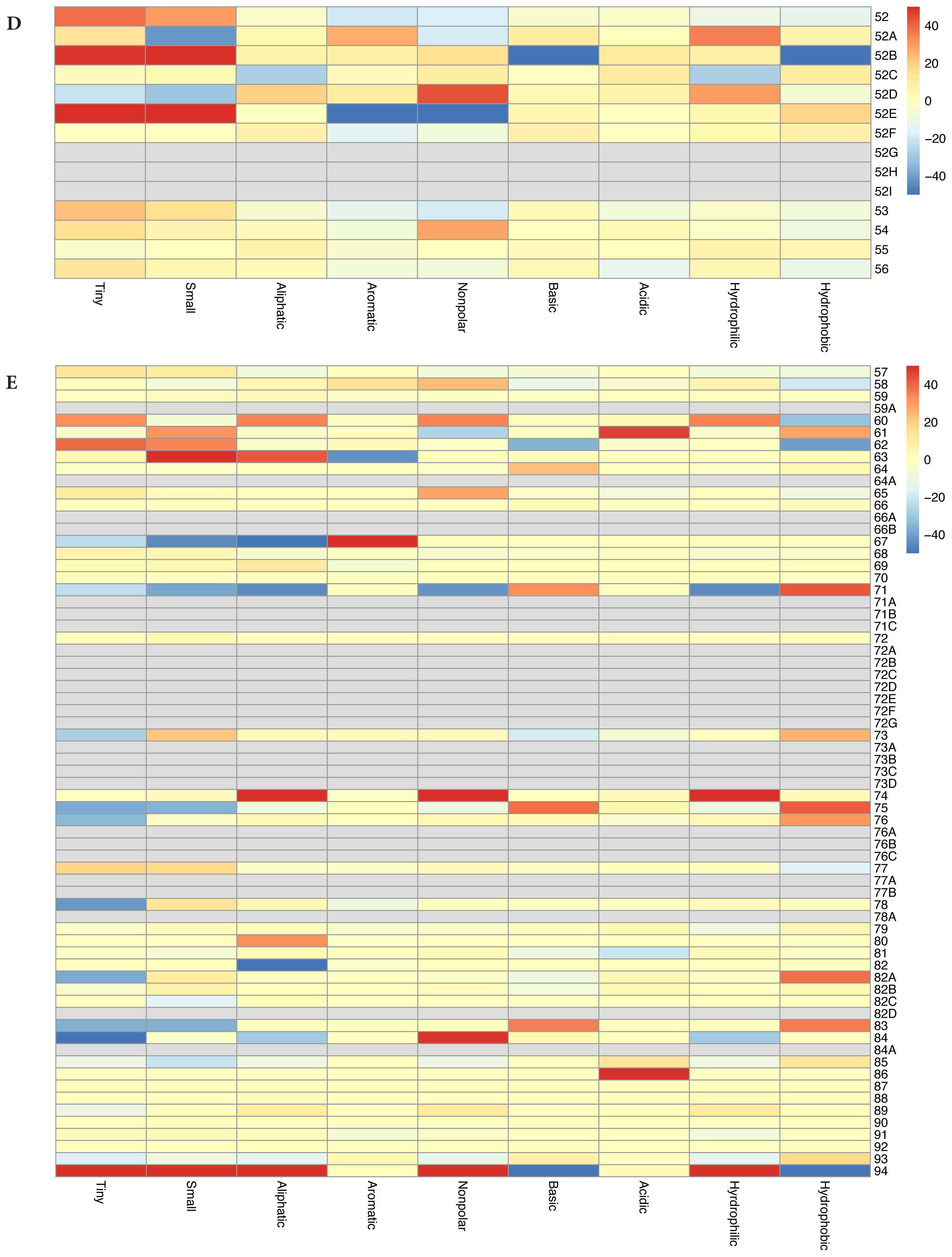

Supplement: Figure S5 — Heatmaps display the differences in physicochemical characteristics of residues between VHs and VHHs. Rows represent individual residues, while columns indicate physicochemical properties of interest as determined in logistic regression analysis. Cell color indicates which antibody format contains a higher percentage of the indicated property in the residue. Red cells indicate a higher property in VHHs, while blue cells indicate a higher property in VHs. Rows in gray indicate alternating positions, where either VHs or VHHs lack the corresponding value, preventing a difference from being calculated. Individual heatmaps show (A) FW1, (B) HCDR1, (C) FW2, (D) HCDR2, (E) FW3, (F) HCDR3, and (G) FW4 residues. The color scale was set to 50 to –50, with higher or lower difference values being set to the set maximum or minimum, respectively. [file tjb-47-06-423s5b.tif]

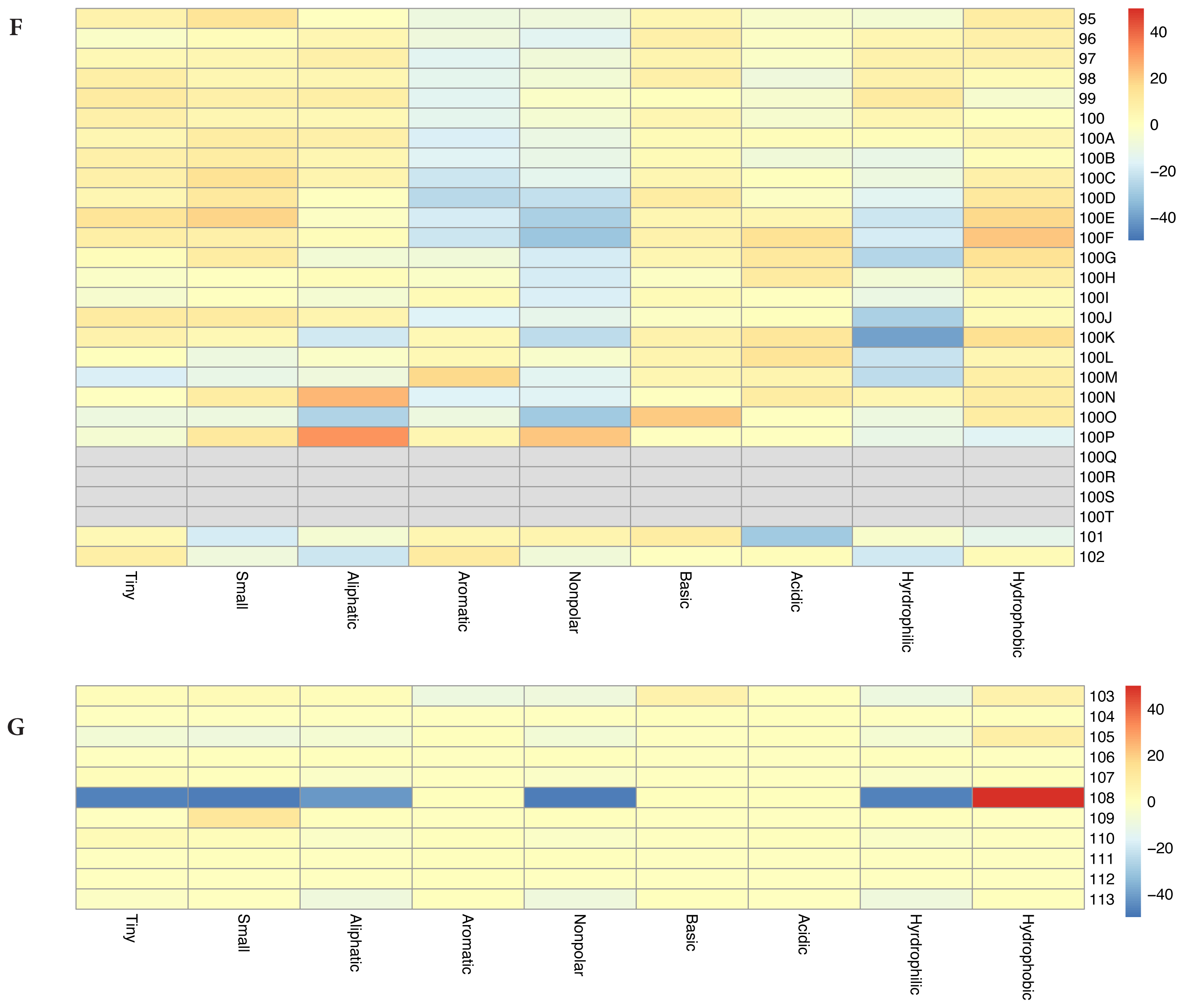

Supplement: Figure S5 — Heatmaps display the differences in physicochemical characteristics of residues between VHs and VHHs. Rows represent individual residues, while columns indicate physicochemical properties of interest as determined in logistic regression analysis. Cell color indicates which antibody format contains a higher percentage of the indicated property in the residue. Red cells indicate a higher property in VHHs, while blue cells indicate a higher property in VHs. Rows in gray indicate alternating positions, where either VHs or VHHs lack the corresponding value, preventing a difference from being calculated. Individual heatmaps show (A) FW1, (B) HCDR1, (C) FW2, (D) HCDR2, (E) FW3, (F) HCDR3, and (G) FW4 residues. The color scale was set to 50 to –50, with higher or lower difference values being set to the set maximum or minimum, respectively. [file tjb-47-06-423s5c.tif]
